# Ruxolitinib combined with corticosteroids for the successful treatment of kawasaki disease-associated macrophage activation syndrome: a case report

**DOI:** 10.3389/fped.2026.1845017

**Published:** 2026-07-28

**Authors:** Xuan Wang, Jiaofeng Bai, Huihui Wang, Yanan Wang, Mayire Ainiwaner, Yaozhu Pan

**Affiliations:** 1 Department of Endocrinology, The 940th Hospital of Joint Logistics Support Force of Chinese People’s Liberation Army, Lanzhou, China; 2 Department of Hematology, The 940th Hospital of Joint Logistics Support Force of Chinese People’s Liberation Army, Lanzhou, China; 3 The First Clinical College of Gansu University of Traditional Chinese Medicine, Lanzhou, China

**Keywords:** corticosteroids, hemophagocytic lymphohistiocytosis, kawasaki disease, macrophage activation syndrome, pediatric, ruxolitinib

## Abstract

**Objective:**

To explore the diagnostic challenges of Kawasaki disease (KD)-associated macrophage activation syndrome (MAS) and the therapeutic value of ruxolitinib combined with corticosteroids.

**Methods:**

We retrospectively analyzed the clinical data of one pediatric patient with KD-associated MAS and conducted sorting and analysis of relevant published literature.

**Results:**

A 14-month-old male infant presented with fever and rash and was diagnosed with severe KD. Despite initial treatment with intravenous immunoglobulin (IVIG) and aspirin, he developed persistent fever, progressive cytopenia, hepatosplenomegaly, hypofibrinogenemia, hyperferritinemia, reduced NK-cell activity, and extremely elevated soluble interleukin-2 receptor (sCD25), fulfilling the HLH-2004 criteria for MAS. He was successfully treated with ruxolitinib plus methylprednisolone, with rapid resolution of fever, recovery of blood counts, and improvement in coagulation and inflammatory markers.

**Conclusions:**

KD-associated MAS is rare but life-threatening. Early recognition of warning signs and prompt initiation of ruxolitinib-based combination therapy can effectively control cytokine storm and improve clinical outcomes.

## 1 Introduction

Kawasaki disease (KD) is the most common acute systemic medium-vessel vasculitis in children (1), predominantly affecting infants and young children, with untreated cases carrying a high risk of coronary artery dilation or aneurysm—the most severe long-term complications (2). Although the majority of patients respond favorably to intravenous immunoglobulin (IVIG) and aspirin, a small subset develops life-threatening secondary complications, among which macrophage activation syndrome (MAS) is one of the most severe (3). MAS belongs to the spectrum of secondary hemophagocytic lymphohistiocytosis (HLH), characterized by uncontrolled activation of T lymphocytes and macrophages, which triggers a massive cytokine storm and subsequent multiple organ dysfunction (4). In recent years, ruxolitinib, a selective Janus kinase (JAK) 1/2 inhibitor, has emerged as a promising targeted therapy for cytokine storm syndromes including MAS; however, clinical reports on ruxolitinib combined with corticosteroids for the treatment of KD-associated MAS remain limited (5). Herein, we report a pediatric patient with severe KD who developed refractory MAS and was successfully treated with ruxolitinib plus methylprednisolone. By sorting and analyzing relevant published literature, we aim to highlight the clinical characteristics, diagnostic pitfalls, and therapeutic value of this regimen, providing valuable evidence for clinical practice.

## 2 Clinical case presentation

The patient was a 14-month-old Han Chinese boy, born full-term via cesarean section with a birth weight of 3.5 kg. His growth and development were consistent with his chronological age, and routine vaccinations were administered in a timely manner. He had no history of allergy, infection, surgery, or chronic disease, and the family history was negative for hereditary or metabolic disorders. He was admitted to our hospital on January 20, 2026, due to 7 days of fever and 6 days of cough accompanied by rash. The fever peaked at 40.0 °C without chills or convulsions. On the second day of fever, a polymorphic erythematous rash appeared on the head and face and spread diffusely to other parts of the body. Prior to admission, antibiotic therapy was administered at a local hospital without any clinical improvement. Physical examination revealed a generalized erythematous rash that blanched on pressure, bilateral conjunctival injection, cracked lips, pharyngeal congestion, and right oral mucosal congestion. No superficial lymphadenopathy was detected, and the liver and spleen were not palpable on admission. Initial laboratory and imaging findings were as follows: white blood cell (WBC) count 17.78 × 10⁹/L, hemoglobin (Hb) 74 g/L, platelet (PLT) count 84 × 10⁹/L; aspartate aminotransferase (AST) 507 IU/L, alanine aminotransferase (ALT) 220 IU/L, albumin (ALB) 25.8 g/L; echocardiography showed bilateral coronary artery dilation (>3.0 mm); abdominal ultrasound revealed no obvious hepatosplenomegaly. Based on these findings, a diagnosis of severe Kawasaki disease was made. The patient initially received standard treatment consisting of aspirin combined with intravenous immunoglobulin (IVIG).Five days after the initiation of initial treatment, the patient experienced clinical deterioration, manifested by persistent fever, progressive rash, abdominal distension, and anorexia. Repeated laboratory examinations showed the following results: WBC count 11.76 × 10⁹/L, Hb 74 g/L, PLT count 28 × 10⁹/L, fibrinogen 0.67 g/L, D-dimer 5.65 mg/L, C-reactive protein (CRP) 26.00 mg/L, interleukin-6 (IL-6) 629.4 pg/mL, natural killer (NK) cell activity 2.7%, soluble interleukin-2 receptor (sIL-2R) 20462.34 U/mL, ferritin 712 μg/L, and triglyceride 3.72 mmol/L. Bone marrow smear examination confirmed the presence of hemophagocytosis, and abdominal ultrasound revealed hepatosplenomegaly and moderate ascites. Based on these clinical manifestations, laboratory findings, and imaging results, the final diagnosis of Kawasaki disease (KD)-associated macrophage activation syndrome (MAS) was established, which met the diagnostic criteria of HLH-2004. The patient was treated with ruxolitinib at a dose of 0.4 mg/kg per day, administered in two divided doses every 12 h. In addition, methylprednisolone was initially administered, followed by a gradual tapering course of oral prednisone. Supportive care was also provided, including fibrinogen transfusion to correct coagulopathy, liver protection therapy, and electrolyte correction. The treatment response was favorable: fever resolved on day 3 after treatment initiation; the rash completely cleared and hepatosplenomegaly improved at 1 week; laboratory markers showed significant recovery at 2 weeks, and the patient was discharged successfully. At the 1-month follow-up, all laboratory parameters returned to normal.

## 3 Discussion

### 3.1 Kawasaki disease and macrophage activation syndrome

Kawasaki disease is the most common acute systemic vasculitis in children, predominantly affecting infants and young children (2). If not promptly treated, it can lead to coronary artery dilation, aneurysm, and even long-term cardiovascular sequelae (3, 4). MAS is a severe, life-threatening complication of KD, belonging to the category of secondary hemophagocytic lymphohistiocytosis (HLH) (5, 6). The incidence of MAS in patients with KD is approximately 1.1%–1.9%, mostly occurring during the acute phase of the disease (7, 8). The pathogenesis of MAS involves uncontrolled activation of T lymphocytes and macrophages, leading to a massive cytokine storm involving interferon-*γ* (IFN-*γ*), IL-6, IL-18, and tumor necrosis factor-α (TNF-α) (9, 10). These proinflammatory cytokines drive widespread tissue damage, cytopenia, coagulopathy, liver failure, and hemophagocytosis (11, 12). Without timely and effective intervention, the mortality rate of MAS can be extremely high (5, 13).

### 3.2 Diagnostic challenges and warning signs

Diagnosing KD-associated MAS is clinically challenging, as many of its clinical and laboratory features overlap with those of severe KD (7, 8). Clinicians should maintain a high index of suspicion for MAS when the following key warning signs are present: persistent or recurrent fever despite standard IVIG and aspirin treatment; unexplained progressive cytopenia; rapidly worsening liver dysfunction; hypofibrinogenemia and hypertriglyceridemia; markedly elevated ferritin and sCD25; decreased NK-cell activity; and the presence of hemophagocytosis on bone marrow examination. The HLH-2004 criteria remain the most widely used diagnostic standard for MAS (5, 6). In the present case, multiple abnormal indicators met the HLH-2004 diagnostic criteria, enabling early identification and intervention.

### 3.3 Rationale for ruxolitinib plus corticosteroids

Corticosteroids are the first-line treatment for MAS; however, some severe cases are refractory to steroid therapy (5, 13). Ruxolitinib, a selective JAK1/2 inhibitor, blocks the signaling pathway of multiple key inflammatory cytokines and has emerged as an effective targeted agent for secondary HLH and MAS (9–11). By inhibiting the activation of the JAK-signal transducer and activator of transcription (STAT) pathway, ruxolitinib reduces the production and release of IFN-*γ*, IL-6, and other proinflammatory cytokines that drive the cytokine storm (11, 12). Recent clinical studies have confirmed the efficacy and safety of ruxolitinib in children with MAS secondary to rheumatic diseases, infection, or KD (8, 10, 13). Combining ruxolitinib with corticosteroids can achieve rapid and synergistic immunosuppressive effects, which is particularly valuable for critically ill pediatric patients (9, 13). Notably, although interleukin-1 inhibitor (anakinra), interleukin-6 receptor antagonist (tocilizumab), and TNF-α inhibitor (infliximab) have been reported as rescue agents for refractory KD and KD-associated MAS in multiple case series (7, 14), ruxolitinib was prioritized as the first-line targeted therapy in this critically ill infant based on three integrated clinical and mechanistic considerations.

Accumulating clinical evidence indicates that cytokine profiles of MAS differ substantially across different pediatric rheumatic diseases, which is a core reference for rational selection of targeted agents (7, 13). Systematic studies on KD-associated MAS have confirmed that, distinct from the relatively single-cytokine dominant phenotype in some other rheumatic disease-related MAS, KD-related MAS presents a multi-cytokine co-activation pattern characterized by simultaneous elevation of IFN-*γ*, IL-6, TNF-α and other inflammatory mediators, which jointly drive the cytokine storm and tissue damage (3, 8). This pathological feature provides a solid mechanistic rationale for prioritizing JAK inhibitors over single-target biologics: by blocking the shared downstream JAK-STAT signaling cascade, JAK inhibitors can simultaneously suppress the biological effects of multiple upstream pro-inflammatory cytokines, rather than only interfering with a single inflammator*y* axis (5, 10, 12).

First, the patient exhibited a multi-cytokine hyperinflammatory storm dominated by excessively elevated IFN-*γ*, IL-6, and soluble IL-2R (sCD25), with serum ferritin reaching 712 μg/L at MAS onset, indicating robust overactivation of the JAK-STAT signaling axis downstream of multiple pro-inflammatory cytokines. Monoclonal biologics such as tocilizumab and anakinra only block single cytokine axes, while ruxolitinib exerts broad inhibitory effects on JAK1/2 to suppress signaling of IFN-*γ*, IL-6, TNF-α simultaneously (15), which better matched the mixed, severe cytokine profile observed in this patient.

Second, the child presented life-threatening critical MAS complicated by severe thrombocytopenia, hypofibrinogenemia, hepatosplenomegaly and ascites after IVIG failure. Rapid suppression of multi-organ hyperinflammation was urgently required; accumulating pediatric HLH/MAS literature confirmed that oral ruxolitinib induces faster resolution of fever and cytopenias compared with single-target injectable biologics in patients with fulminant secondary HLH (16, 17).

Third, practical administration advantages favored ruxolitinib for this 14-month-old infant: oral twice-daily dosing avoided repeated subcutaneous or intravenous injections required by anakinra, tocilizumab and infliximab, reducing procedural distress and infection risk in a critically unwell young child.

Notably, anti-TNF agents like infliximab are primarily recommended for IVIG-refractory KD without overt MAS (18), with limited high-quality evidence for fulminant KD-MAS complicated by severe coagulopathy and bone marrow hemophagocytosis, which further limited its priority in this case.

To intuitively demonstrate the dynamic changes of five core indicators — C-reactive protein (CRP), ferritin, fibrinogen (FIB), platelet count (PLT) and liver enzymes [aspartate aminotransferase (AST), alanine aminotransferase (ALT)] — across pre-MAS onset, MAS diagnosis, and post-ruxolitinib combination therapy stages, and to further verify the aforementioned therapeutic effects, all key serial laboratory results are summarized in Table 1.

**Table 1:** Serial changes of core laboratory biomarkers in KD-MAS throughout the clinical course.

Stage	Time point	PLT (×10⁹/L)	Fibrinogen (FIB, g/L)	CRP (mg/L)	Ferritin (*μ*g/L)	Liver enzymes (AST/ALT, IU/L)
Pre-MAS (initial KD, pre-IVIG)	Admission day 1	84	Not tested	Not tested	Not tested	507/220
At MAS diagnosis (5 days post-IVIG, pre-targeted therapy)	Illness day 12	28	0.67	26.00	712	Persistently elevated
Post ruxolitinib + steroid therapy day 3	Targeted therapy day 3	47	1.32	11.40	426	189/96
Post ruxolitinib + steroid therapy week 1	Targeted therapy day 7	106	2.15	3.70	168	63/31
Post ruxolitinib + steroid therapy week 2 (discharge)	Targeted therapy day 14	213	2.68	0.82	74	41/22
1-month outpatient follow-up	Illness day 47	276	2.95	＜0.5	36	Normal

### 3.4 Prognosis and clinical implications

The prognosis of MAS is closely associated with early diagnosis and timely treatment (5, 7). Delayed intervention often leads to disseminated intravascular coagulation, multiple organ failure, and even death (6, 8). Long-term follow-up is necessary to monitor coronary artery lesions and the recurrence of MAS. This case report indicates that ruxolitinib combined with corticosteroids is an effective rescue regimen for KD-associated MAS. Clinicians should maintain a high level of vigilance for MAS in patients with severe KD and initiate targeted immunotherapy promptly when MAS is suspected.

## 4 Conclusion

We reported a case of KD-associated MAS that was successfully treated with ruxolitinib combined with corticosteroids, and sorted and analyzed relevant published literature. KD-associated MAS is a rare but life-threatening complication. Early recognition of its warning signs and prompt initiation of ruxolitinib-based combination targeted therapy are essential to control the cytokine storm and improve patient survival. This therapeutic regimen provides a valuable reference for clinical practice in the management of KD-associated MAS.

## Data Availability

The raw data supporting the conclusions of this article will be made available by the authors, without undue reservation.
